# Subcellular Spatial Transcriptomes: Emerging Frontier for Understanding Gene Regulation

**DOI:** 10.1101/sqb.2019.84.040352

**Published:** 2020-06-01

**Authors:** Furqan M. Fazal, Howard Y. Chang

**Affiliations:** 1 Center for Personal Dynamic Regulomes, Stanford University, Stanford, California 94305, USA; 2 Howard Hughes Medical Institute, Stanford University School of Medicine, Stanford, California 94305, USA

## Abstract

RNAs are trafficked and localized with exquisite precision inside the cell. Studies of candidate messenger RNAs have shown the vital importance of RNA subcellular location in development and cellular function. New sequencing- and imaging-based methods are providing complementary insights into subcellular localization of RNAs transcriptome-wide. APEX-seq and ribosome profiling as well as proximity-labeling approaches have revealed thousands of transcript isoforms are localized to distinct cytotopic locations, including locations that defy biochemical fractionation and hence were missed by prior studies. Sequences in the 3′ and 5′ untranslated regions (UTRs) serve as “zip codes” to direct transcripts to particular locales, and it is clear that intronic and retrotransposable sequences within transcripts have been co-opted by cells to control localization. Molecular motors, nuclear-to-cytosol RNA export, liquid-liquid phase separation, RNA modifications, and RNA structure dynamically shape the subcellular transcriptome. Location-based RNA regulation continues to pose new mysteries for the field, yet promises to reveal insights into fundamental cell biology and disease mechanisms.

A eukaryotic cell is highly organized, with biomolecules localizing to specific regions of the cell that are integral to their function. For more than three decades, evidence has been accumulating to suggest that the RNAs for thousands of genes show pronounced subcellular localization, and that this localization is an essential mechanism for post-transcriptional regulation. RNA localization influences RNA folding, editing, splicing, degradation, translation, binding partner, catalytic activity, and even the fate of the protein that is encoded. Some of the earliest experiments examining the localization of messenger RNAs (mRNAs) were performed in *Xenopus* and *Drosophila* eggs, and were followed by similar demonstrations in yeast, mammalian neurons, and in developing *Drosophila* embryos. Such studies have revealed that sequences within (i.e., *cis* elements) RNAs, also termed “zip codes,” direct the localization of mRNAs, typically by recruiting proteins (i.e., *trans* factors).

In this review, we summarize the history of mRNA localization studies and focus on exciting new developments in the last decade to track the localization of thousands of transcripts within cells using either sequencing- or imaging-based approaches. We identify how new techniques are starting to systematically dissect the *cis* and *trans* regulators of RNA localization. Although it now appears that RNA subcellular localization is the norm rather than the exception for both coding and noncoding RNAs (Wilk et al. 2016) and is broadly conserved evolutionarily (Benoit Bouvrette et al. 2018), our understanding of the extent, importance, and regulation of subcellular spatial transcriptomics continues to be limited. Furthermore, the relevant techniques toolkit for such RNA studies lags behind those developed for subcellular spatial proteomics, for which we have detailed information for more than 10,000 human protein-coding genes with subcellular resolution (Uhlen et al. 2010; Thul et al. 2017) across many tissues (Uhlen et al. 2015). In contrast, even today we do not have a good map or atlas of RNA subcellular localization, although promising new technological developments (Chen et al. 2015; Shah et al. 2016; Fazal et al. 2019) are making such milestones within reach.

## BRIEF HISTORY OF RNA LOCALIZATION STUDIES

**Figure 1.**
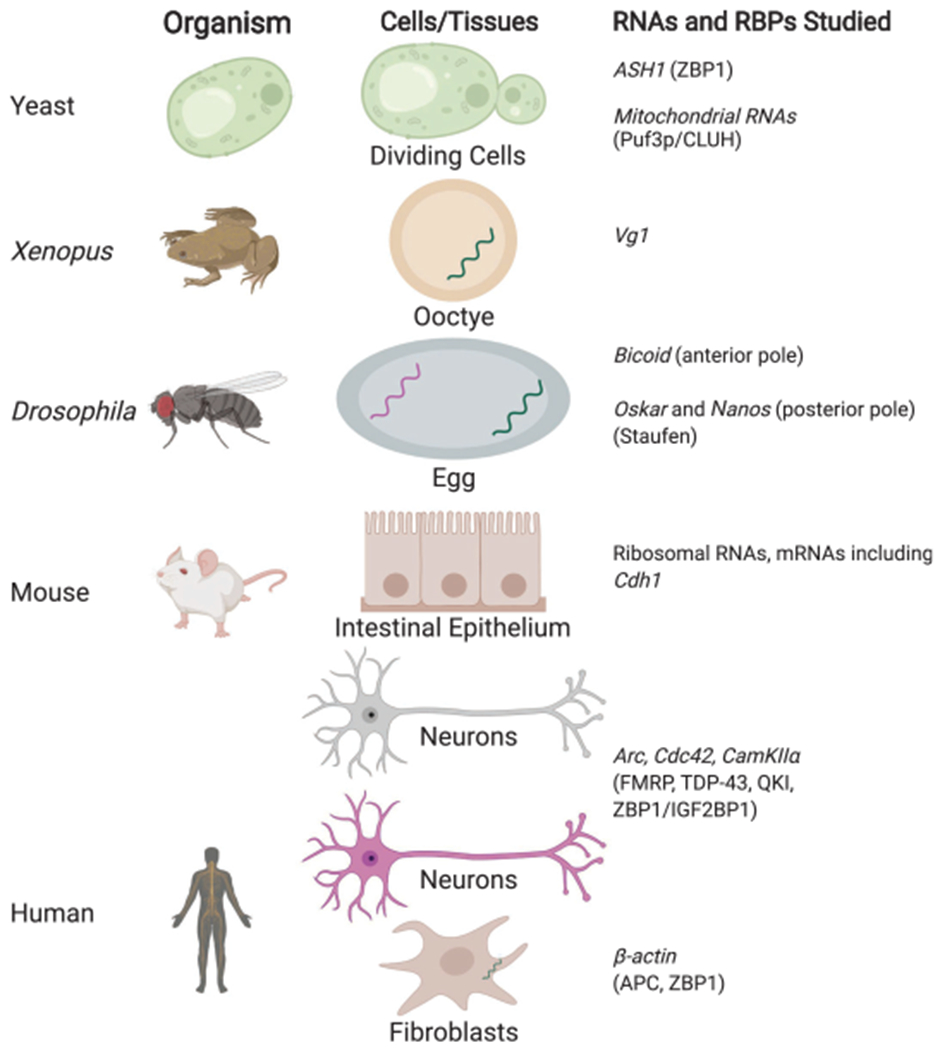
Model systems to study RNA localization. RNA-binding proteins involved are shown in parentheses.

Early studies in RNA localization focused on easy-to-image cells such as the relatively large *Xenopus* (Rebagliati et al. 1985; Weeks and Melton 1987) and *Drosophila* eggs. Such initial studies established mRNA localization as a way to regulate protein expression and have shown that sequences within the transcript, particularly in the 3′ untranslated region (UTR), can direct localization of transcripts; these findings have since been extended to mammalian systems. For example, the *Vg1* mRNA in *Xenopus* was found to localize to one (the vegetal) pole, whereas the *bicoid* mRNA in *Drosophila* egg cell was shown to localize to the anterior pole and to require the protein Staufen for localization (Fig. 1; St Johnston et al. 1991).

In mammals, one of the most well-studied transcripts is *β-actin* mRNA, which localizes to the leading edge of chicken embryo fibroblasts and to the growth cones of developing neurons. *β-actin* has been shown to contain a 54-nucleotide (nt) zip code region in the 3′ UTR that is essential for localization (Kislauskis et al. 1994) in a translation-independent manner. This localization is, in turn, regulated by the protein factors IGF2BP1 (ZBP1) and ZBP2. ZBP1 controls local translation of β-actin by sequestering the transcript until it reaches the periphery of the cell, where the phosphorylation of ZBP1 releases the mRNA and permits its translation (Huttelmaier et al. 2005). Other RNAs have similarly been shown to localize to cellular protrusions and to require adenomatous polyposis coil (APC) protein (Mili et al. 2008; Baumann et al. 2020). Likewise, the protein fragile X mental retardation protein (FMRP) functions as a translational regulator of localized RNAs in many systems, including neurons and fibroblasts (Mili et al. 2008). FMRP functions by binding to and repressing the translation of mRNAs and is mediated by recognition of RNA secondary structure. Upon reaching their destination, FMRP release of the RNAs triggers local translation, as in the case of axons. Findings from many studies have converged on the hypothesis that the mRNAs are transported along with retinol-binding proteins (RBPs) in the cytosol as translationally repressed RNA granules (Anderson and Kedersha 2006). Supporting studies have shown that the cytoskeleton and its associated molecular motors play an integral role in this mRNA transport (Wang et al. 2016).

**Figure 2.**
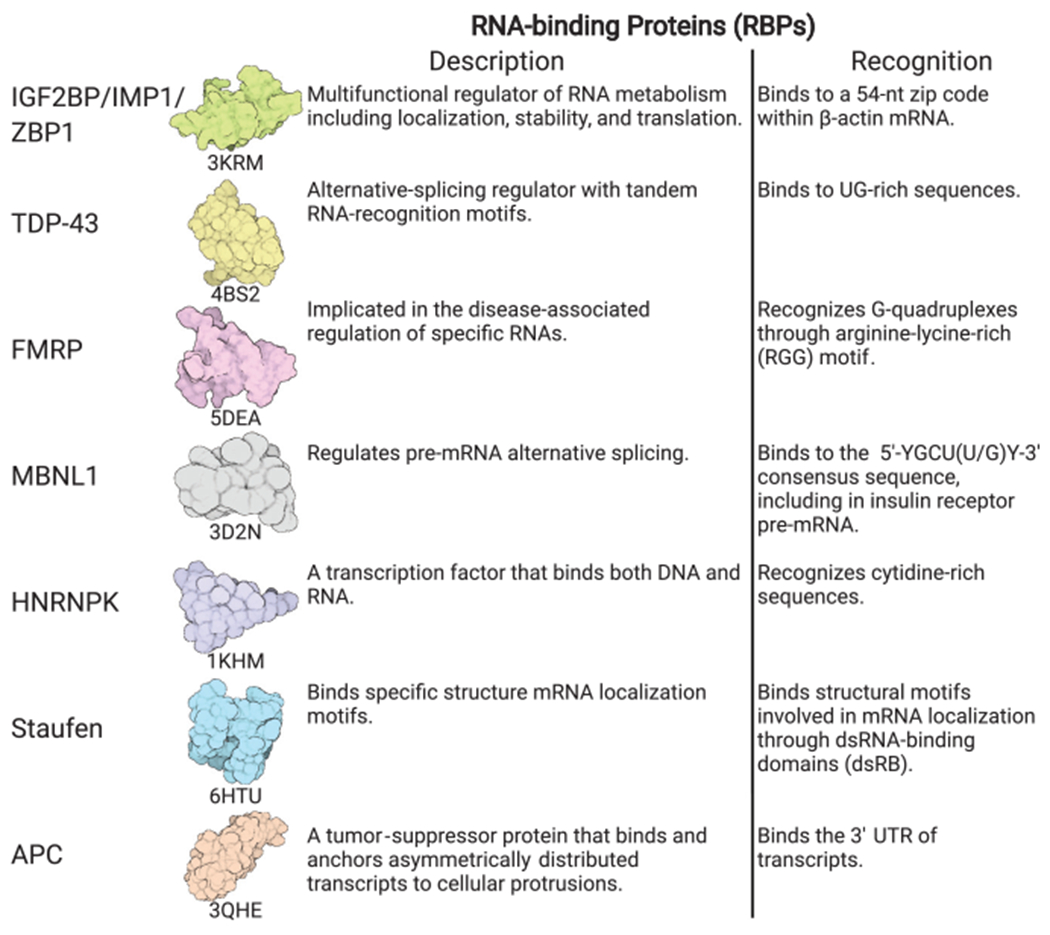
Some representative RNA binding proteins implicated in mRNA localization. Structures are generated from Protein Data Bank (PDB) entries.

The lessons learned to dissect *β-actin* mRNA transport have since been extended to other mammalian systems, particularly neurons that are ideal systems to study localization defects because of the vast distances metabolites need to be transported. Neurons need to coordinate functions between the cell nucleus and the axons and dendrites, which can be >1 m apart. Neurons also need to dynamically regulate their proteomes in response to changing environments, and it is now clear that local translation of mRNAs in dendrites is widespread and essential. It is now thought that RNA localization is the primary determinant of the proteome of neurites, rather than transport of corresponding proteins (Zappulo et al. 2017). Further-more, many essential RBPs whose processing is dysregulated in neuronal disorders have been shown to bind hundreds of RNAs and to be involved in their localization. Two such RBPs are FMRP, whose loss of function results in fragile X syndrome and autism, and TDP-43, whose dysregulation is associated with amyotrophic lateral sclerosis (ALS) (Neumann et al. 2006; Sreedharan et al. 2008). TDP-43, which regulates RNA metabolism through many mechanisms, will form cytoplasmic messenger ribonucleoprotein (mRNP) granules that undergo microtubule-dependent transport in neurons (Fig. 2; Alami et al. 2014).

## MECHANISMS OF RNA LOCALIZATION

### Role of *cis* Elements, including Retrotransposable Elements and Features in the 3′ UTR

Studies focusing on specific RNAs such as *β-actin* have revealed that mRNAs can localize to subcellular locales independent of translation and are guided by internal zip code sequences, particularly in 3′ UTRs. Surprisingly, even smaller RNAs such as microRNAs (miRNAs) can contain sequence elements within that direct them to subcellular locales (Hwang et al. 2007), as do many long noncoding RNAs (lncRNAs) (Batista and Chang 2013). However, for the vast majority of transcripts, the zip codes responsible for localization to specific organelles and biological condensates remain unknown, although newly developed transcriptome-wide approaches are laying the foundation for identifying more *cis*- localization elements. For example, in neurons, hundreds of genes, including *Cdc42*, have transcript isoforms that localize differently between neurites and the soma based on sequence differences in 3′ UTRs (Ciolli Mattioli et al. 2019). Similarly, the endoplasmic reticulum (ER) is known to recruit transcripts directly in a translation-independent matter (Pyhtila et al. 2008), and a recent transcriptome-wide study has identified a sequence termed SECRETE that can recruit mRNAs encoding secretory/membrane proteins to the ER. The SECRETE sequence, comprising a ≥10-nt triplet repeat, occurs in both prokaryotes and eukaryotes (Cohen-Zontag et al. 2019).

**Figure 3.**
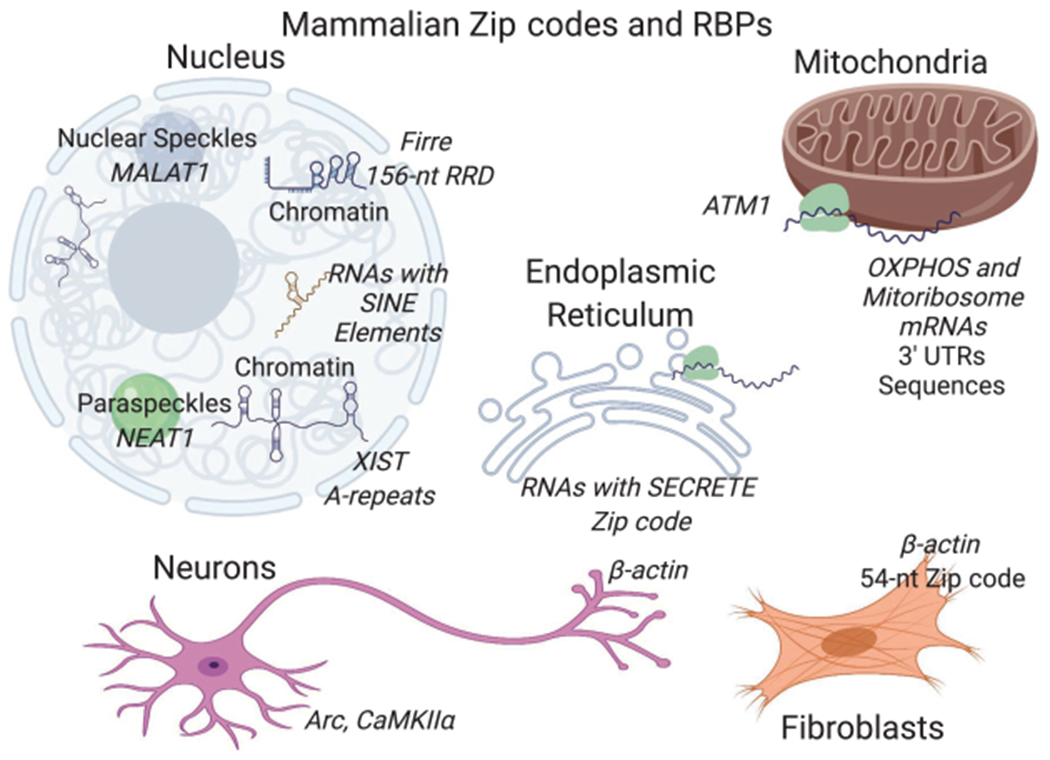
Examples of locales with localized RNAs and associated zip codes.

A robust approach to identify zip codes within transcripts, which has been particularly fruitful for lncRNAs, is to identify a transcript(s) that localizes to a specific locale and then to systematically test whether sequences within are necessary and sufficient to direct localization (Fig. 3). Such an approach has identified an ~600-nt region in human cells that is required for localization of the lncRNA *MALAT1* to nuclear speckles (Miyagawa et al. 2012). Similarly, sequences within the lncRNA *Xist* called A-repeats, located near the 5′ end, are responsible for localization to the nuclear periphery (Wutz et al. 2002), likely secondary to the ability of this RNA element to induce facultative heterochromatinization (Chaumeil et al. 2006). Another lncRNA *Firre* has a 156-nt repeating RNA domain (RRD), recognized by the protein hnRNPU, that aids in localizing it to chromatin (Hacisuleyman et al. 2014); hnRNPU binding is also required for the proper localization of *Xist* (Hasegawa et al. 2010). Recently several studies, including computation and experimental approaches, have revealed that sequences derived from transposable elements, which are present in many mRNAs and lncRNAs, contribute to the nuclear retention of many lncRNAs (Carlevaro-Fita et al. 2019). Although such studies show the widespread occurrence of zip code sequences, systematic high-throughput experiments are needed to identify the *cis* elements necessary for the observed extensive RNA subcellular localization transcripts.

### Protein Factors That Interact with mRNAs and lncRNAs

RNA localization is thought to be orchestrated by RNA-binding proteins that can recognize sequence motifs or RNA structural features, including single-stranded regions or stem loops. Although we know of a few RBPs mediating localization, including Staufen and Puf3, how the cell coordinates the localization of thousands of transcripts remains poorly understood. In the case of Staufen, binding to double-stranded sequences within maternal RNAs (St Johnston et al. 1992) results in their subcellular localization, whereas for the Puf3 protein binding to a sequence motif (Zhu et al. 2009) within some nuclear-encoded RNAs results in their recruitment to the mitochondria in yeast (Saint-Georges et al. 2008; Gadir et al. 2011). Future work on mapping RNA–protein interactions (Ramanathan et al. 2019) will likely be crucial in discovering RBPs essential for localization.

### Active Transport and the Role of Molecular Motors

**Figure 4.**
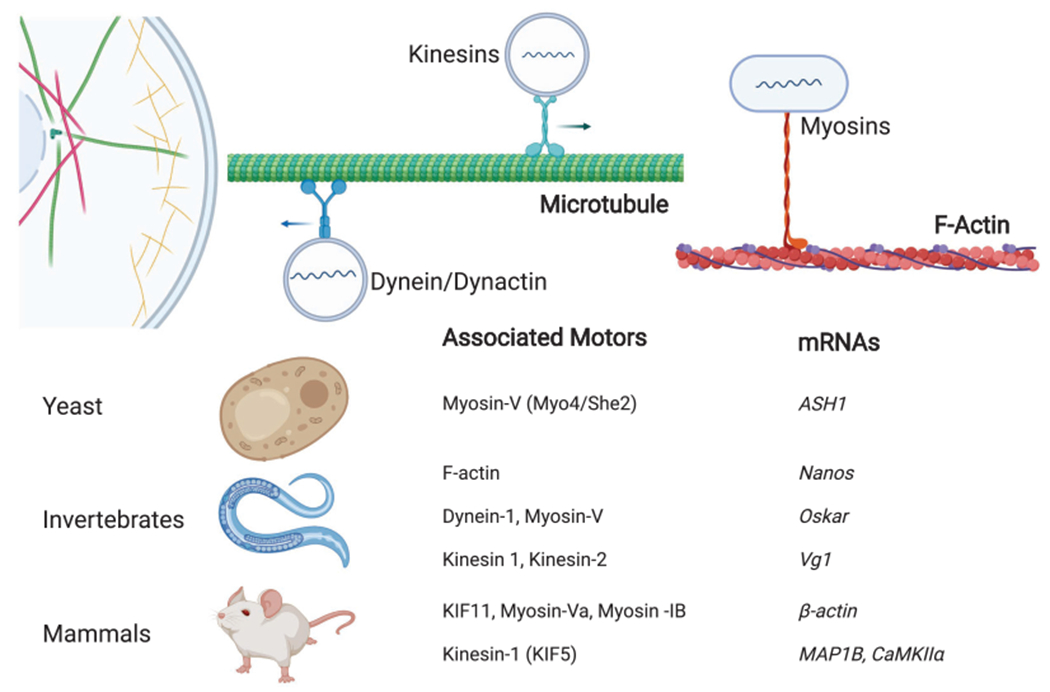
Molecular motors implicated in RNA transport.

Many studies suggest mRNA localization in the cytosol is facilitated by the underlying cytoskeleton network, although the relative contributions of individual players remain unclear. However, we do know that molecular motors operating on microtubules as well as actin filaments participate in RNA transport (Fig. 4; Maday et al. 2014), including myosin motors that walk on actin filaments and kinesins and cytoplasmic dynein that move on microtubules. For example, early studies established that the localization of *oskar* mRNA in *Drosophila* oocytes to the posterior pole requires the cytoskeleton (Erdelyi et al. 1995), with subsequent studies implicating kinesin-1 (Zimyanin et al. 2008). Similar studies of other RNAs in yeast have implicated myosin V (Bertrand et al. 1998).

Furthermore, this RNA localization process can be dynamically regulated through active transport, as shown in intestinal epithelial cells where mRNAs strongly localize (Moor et al. 2017). The RNA, packaged in RNPs, can be transported bidirectionally along microtubules by plus-end-directed kinesins (Kanai et al. 2004) and minus-end-directed dynein motors (Hirokawa et al. 2010). Kinesins typically transport RNAs toward the cell periphery, where-as dynein transports RNAs toward the cell center (retrograde transport). However, how the different motors cooperate is unclear, and in fact, different motor types are known to engage cargo and participate in tug-of-war coordination (Hancock 2014). A recent transcriptome-wide study has confirmed that hundreds of transcripts rely on microtubule-based transport to get their cytosolic destinations (Fazal et al. 2019), and continued progress is being made in understanding the transport of RNAs, as shown in reconstituted in vitro systems (Baumann et al. 2020) and inside living cells (Krauss et al. 2009).

### Splicing, Intron Retention, and Nuclear Export

The nucleus of a eukaryotic cell is enveloped by a double lipid bilayer that serves as the gateway for mRNAs exiting to the cytosol. The export of RNAs through the nuclear pore complexes (NPCs) spanning the envelope has been extensively studied (Muller-McNicoll and Neugebauer 2013; Katahira 2015), with translocation through the pore thought to be diffusive, and with only a fraction (one-third or less) of mammalian mRNAs that interact with the NPC eventually exiting (Ma et al. 2013). Importantly, this export process can vary depending on the type of RNA species (mRNAs, ribosomal RNAs [rRNAs], micrmiRNAs, transfer RNAs [tRNAs], etc.) in question (Muller-McNicoll and Neugebauer 2013; Katahira 2015). Furthermore, mRNAs can move bidirectionally through the pore, and not all pores are created equally (Grunwald and Singer 2010; Siebrasse et al. 2012). The NPCs are hypothesized to show considerable heterogeneity (Colon-Ramos et al. 2003), with specialized NPCs mediating the transport of mRNAs from distinct genomic loci with nuclei to specific regions of the cytosol and, therefore, optimizing nuclear export and facilitating subsequent translation (Brown and Silver 2007).

Within the nucleus, splicing has a profound influence on nuclear export (Kim-Ha et al. 1993; Hachet and Ephrussi 2004), with pre-mRNAs recruiting splicing factors along with the conserved mRNA export machinery (TREX, transcription/export complex). TREX is recruited to the 5′ end of transcripts and accounts for the export of mRNAs through the pore (Cheng et al. 2006). Likewise, the deposition of exon-junction complex (EJC) during splicing is essential for the localization of developmentally important transcripts (Braunschweig et al. 2013), including *oskar* mRNA in *Drosophila* (Ghosh et al. 2012). Alternative splicing provides yet another opportunity for the cell to influence RNA localization, as has been shown recently where isoform-specific localization to neurites is guided by alternative last exons (ALEs) (Taliaferro et al. 2016). Furthermore, partial splicing of transcripts results in their nuclear retention, which partially explains why many lncRNAs that are substantially less-efficiently spliced relative to mRNAs are nuclear (Zuckerman and Ulitsky 2019). By retaining some introns (“detained introns”) in polyadenylated transcripts that are only excised before export, cells use nuclear retention in mRNAs and a constant nuclear-export rate to reduce cytoplasmic gene expression noise due to bursty transcription-related noise (Bahar Halpern et al. 2015). Such detained introns are widespread, enriched in UTRs and noncoding RNAs, and thought to functionally tune transcriptomes (Braunschweig et al. 2014).

## MODERN APPROACHES TO STUDY RNA LOCALIZATION

### Tracking Single RNAs

**Figure 5.**
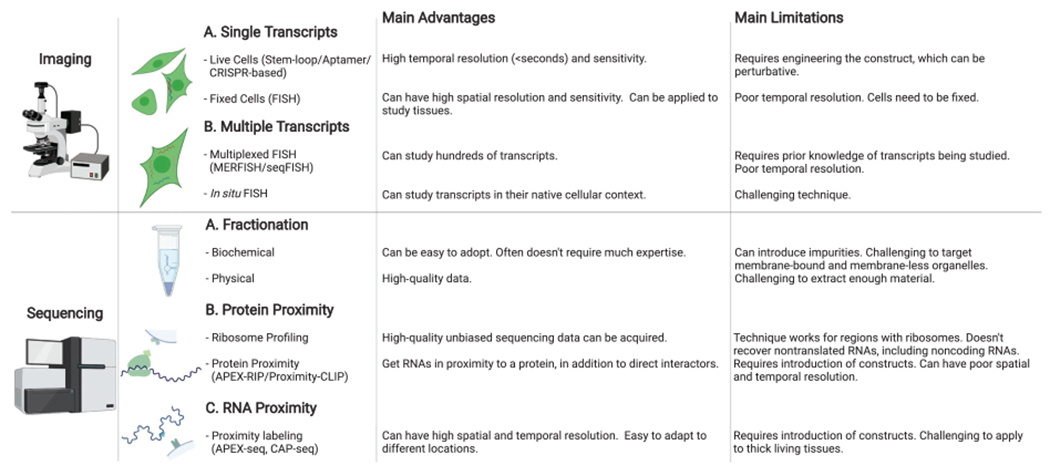
Techniques to study RNA localization.

Currently, there are two general approaches to map RNA subcellular localization: imaging- and sequencing-based. Imaging-based approaches over the last two decades have yielded insights into the dynamics of single RNAs in cells, revealing their complicated history. One of the early studies focused on the dynamics of *ASH1* mRNA in yeast, and its 3′ UTR localization using the MS2 RNA-hairpin system (Fig. 5; Bertrand et al. 1998). Since then, the MS2 system has been optimized and applied extensively to study mRNA localization and transcription in living cells (Darzacq et al. 2007), and complementary approaches have been developed (Wu et al. 2016, 2019; Braselmann et al. 2018; Chen et al. 2019a; Wan et al. 2019) to study localization and local translation. Similar studies have revealed the intricate dynamics of nuclear pore mRNA export (Grunwald and Singer 2010; Siebrasse et al. 2012; Chen et al. 2017), and the trafficking of mRNAs to membrane-less organelles (MLOs) such as stress granules (Nelles et al. 2016). In addition to live-cell imaging, in situ hybridization approaches (Lawrence et al. 1989), which have evolved to use fluorescent in situ hybridization (FISH) labeling (Femino et al. 1998), provide complementary information with routine single-molecule sensitivity (Raj et al. 2008). The FISH-based approach has recently been extended to study RNA localization for hundreds of thousands of RNAs simultaneously, as discussed below.

### Transcriptome-Wide Imaging Technologies

An exciting development in the field is the new approaches that finally enable imaging of hundreds and even thousands of RNAs within fixed cells. An early study was based on in situ RNA sequencing using complementary DNA amplicons, which permitted thousands of RNAs to be simultaneously interrogated (Lee et al. 2014). However, although this technology is promising (Ke et al. 2013; Lee et al. 2014) and improvements continue to be made (Fürth et al. 2019), so far this challenging approach has not been widely adopted. Instead, visualizing many RNAs using sequential FISH is at the forefront of high-throughput localization studies, and two groups have mainly advanced this approach. In one iteration, called MERFISH developed by the Zhuang laboratory, the locations of RNAs in fixed cells are interrogated by performing sequential FISH through multiple rounds of hybridization of DNA oligonucleotides (“oligos”) to the complementary RNAs of interest. This sequential approach uses an error-correcting scheme to design and select for hybridization oligos, such that some errors in the binding of DNA oligos to the complementary RNA molecules can be tolerated and correctly decoded. Although the high density of RNAs in a cell puts a limit on how many transcripts can be resolved and their relative abundances (Chen et al. 2015), in practice, hundreds to thousands of transcripts in individual cells can be interrogated. Further advances, including the integration of other techniques such as expansion microscopy, have further aided throughput (Xia et al. 2019). Recently the MERFISH approach has also been extended to carry out phenotypic screening in cells (Emanuel et al. 2017), as shown by a study identifying positive and negative regulators of the nuclear-speckle localization of the lncRNA *MALAT1* (Wang et al. 2019a).

The second sequential FISH approach, advanced by Cai and coworkers, called SeqFISH (Lubeck et al. 2014; Shah et al. 2016), allows multiplexed imaging of hundreds of genes through signal amplification and error-correction schemes, similar to MERFISH. Excitingly, SeqFISH facilitates mapping the subcellular localization of thousands of RNAs, including nascent transcripts (Shah et al. 2018) and splice isoforms. However, the limitations of both MERFISH and SeqFISH include working with fixed and not live cells. Furthermore, unlike sequencing that can be unbiased, the techniques require prior knowledge of the transcripts being targeted, as oligos can be designed to image those transcripts. In the near term, the unique advantage of imaging-based approaches in simultaneously interrogating many cells makes them especially well suited in exploring RNA heterogeneity across cells and in tissues (Moffitt et al. 2018), thereby distinguishing cell-types based on the RNAs they express (Eng et al. 2019).

### Transcriptome-Wide Sequencing Technologies

The advent of next-generation sequencing technologies has ushered in a new revolution in biology, including in the investigation of RNA localization. Biochemical fractionation protocols coupled with RNA sequencing have, for example, been applied to study nuclear-versus-cytosol RNA dynamics (Djebali et al. 2012; Benoit Bouvrette et al. 2018) and to determine RNAs being actively translated through polyribosome profiling. In recent years, fractionation protocols have also been developed to determine the RNAs in challenging locations such as the membraneless nucleolus and stress granules (Khong et al. 2017). Likewise, physical/mechanical separation of long neuronal cells, typically through microdissection (Cajigas et al. 2012), has been productive in determining their transcriptomes. Other techniques such as laser capture microscopy (LCM) have also enabled careful dissection of both single cells and subcellular locations (Nichterwitz et al. 2016) within and been applied to study rapid changes in RNA localization in mouse intestinal epithelial cells in response to food gradients (Moor et al. 2017).

An innovative approach to study the RNAs in cytosolic locales such as the endoplasmic reticulum membrane (ERM) and outer mitochondrial membrane (OMM) has been through proximity-specific ribosome profiling, in which ribosomes in specific locations undergo proximity biotinylation (Roux et al. 2012). These ribosomes with a biotin tag can subsequently be isolated through streptavidin-biotin pulldown, and the RNAs they are bound to and translating profiled by sequencing them. Such ribosome profiling experiments have revealed the RNAs bound to ribosomes in the ER in yeast and humans (Jan et al. 2014) and at the outer surface of the mitochondria in yeast (Williams et al. 2014; Costa et al. 2018).

Despite these existing sequencing technologies, many critical locations within the cell, including membrane-bound and membrane-less organelles, continue to be difficult, if not impossible, to interrogate. Furthermore, unlike live-cell-imaging approaches, these sequencing-based approaches are generally not well suited to study the dynamics of transcript localization. However, a new approach discussed below using proximity labeling of RNAs in living cells provides an opportunity to investigate RNA subcellular spatial dynamics (Fazal et al. 2019; Padron et al. 2019), albeit currently at a bulk rather than single-cell scale.

### Subcellular Transcriptomics through Proximity Labeling

A recent approach to determine the RNAs at subcellular locales, called APEX-seq, yields an unbiased transcriptome that can be applied to study membrane-less and membrane-bound organelles. APEX-seq leverages an engineered enzyme called APEX2 (ascorbate peroxidase, version 2) that can be targeted to specific cellular locales by fusing it to a protein or peptide that is known to localize to the desired location. Upon providing the reagents biotin-phenol and hydrogen peroxide, APEX2 generates biotin-phenoxy radicals that result in the spatial tagging of nearby metabolites within cells with a biotin tag (Rhee et al. 2013). For example, when plasmids containing APEX2, which itself is around the size of green fluorescent protein (GFP), are fused to the nuclear localization sequence (NLS) and introduced into cells, APEX2 localizes to the nucleus and permits tagging of metabolites there. These metabolites include proteins (Rhee et al. 2013), RNAs, DNA, and small molecules; in APEX-seq, the labeled RNAs are isolated and enriched for using streptavidin-biotin pulldown, followed by RNA sequencing. Excitingly, APEX RNA labeling can achieve high spatial (~10-nm) and temporal (~1-min) resolution in almost any location of interest, including in MLOs such as the nucleolus (Fazal et al. 2019) and stress granules (Markmiller et al. 2018), as well as the membrane-bound ER (Kaewsapsak et al. 2017) and outer mitochondrial membrane (Fazal et al. 2019). Furthermore, APEX-based approaches have been applied to different model systems, including in mice, worms, and flies, as well as in cultured neurons (Hung et al. 2016). Likewise, fusing APEX to dCas9 (Gao et al. 2018; Myers et al. 2018; Qiu et al. 2019) allows targeting APEX to any genomic locus and obtaining the interacting proteins and RNAs.

In the initial demonstrations of obtaining subcellular RNAs using proximity labeling, APEX was used to label proteins, which were then cross-linked with RNAs nearby. These biotin-labeled proteins were then enriched by streptavidin-biotin pulldown, and the cross-linked RNAs were released and sequenced. Using this cross-linking approach, called APEX-RIP (Kaewsapsak et al. 2017) and proximity-CLIP (Benhalevy et al. 2018), APEX labeling has been used to determine the RNAs in the cytosol, nucleus, and mitochondria. In APEX-RIP, formaldehyde cross-linking is performed, whereas in proximity-CLIP UV cross-linking and metabolic labeling is used to improve specificity.

In contrast, the more straightforward APEX-seq approach entailing direct RNA labeling (Zhou et al. 2019) has generated subcellular transcriptomes of many organelles in human cells (Fazal et al. 2019; Padron et al. 2019). By targeting APEX to multiple subcellular locales in the nucleus and cytosol, APEX-seq has revealed that thousands of RNAs show robust subcellular localization (Fazal et al. 2019). Independently, Ingolia and coworkers have used APEX-seq to examine RNAs in proximity to the 7-methylguanosine (m^7^G) cap-binding protein eIF4E1, while also obtaining subcellular proteomic information. In addition, changes in RNA localization upon heat shock and stress granule assembly on the timescale of minutes were tracked (Padron et al. 2019).

APEX-seq (Fazal et al. 2019) has revealed that the RNA transcripts for thousands of genes localize to specific locales within cells, including in the nucleolus, nuclear lamina, nuclear pore, OMM, and ERM. Moreover, APEX-seq detected many transcripts with distinct isoforms showing differential subcellular localization. In addition to providing a map of subcellular RNA localization, APEX-seq has confirmed the role of the nuclear pore in mRNA surveillance and shown that the location of mature RNA transcripts within the nucleus is connected with the underlying genome architecture. For example, transcripts found at the nuclear lamina are enriched for genes found in DNA lamina-associated domains (LADs), as well as transcripts containing retrotransposable elements such as the short interspersed nuclear elements (SINEs) and long interspersed nuclear elements (LINEs). APEX-seq also revealed two modes of mRNA localization to the OMM: ribosome-dependent (i.e., requiring translation) and RNA-dependent. Transcripts coding for mitochondrial proteins that localize to the OMM independent of translation were found to have shorter 3′ UTRs and shorter poly(A) tails. RNA localization to the OMM depends on active transport, as shown by time course experiments showing mislocalization of transcripts within minutes of adding nocodazole, a microtubule depolymerizer.

APEX-seq, in conjunction with approaches to identify proteins interacting with specific RNAs (Chu et al. 2015; Ramanathan et al. 2018,^2019^; Mukherjee et al. 2019; Han et al. 2020), is likely to emerge as a powerful approach to identify both localized RNAs and their corresponding RBP partners. Likewise, new approaches to spatial tagging of RNAs are continually being invented. One such method is based on spatially restricted nucleobase oxidation, which uses localized fluorophores (Li et al. 2017). Another approach uses an enzyme to add uridine residues to RNAs in specific locations in *Caenorhabditis elegans*, including in the mitochondria and ER (Medina-Muñoz et al. 2019). A third approach, called CAP-seq, uses light-activated, proximity-dependent photo-oxidation of RNA (Wang et al. 2019b).

### Machine-Learning Approaches

**Figure 6.**
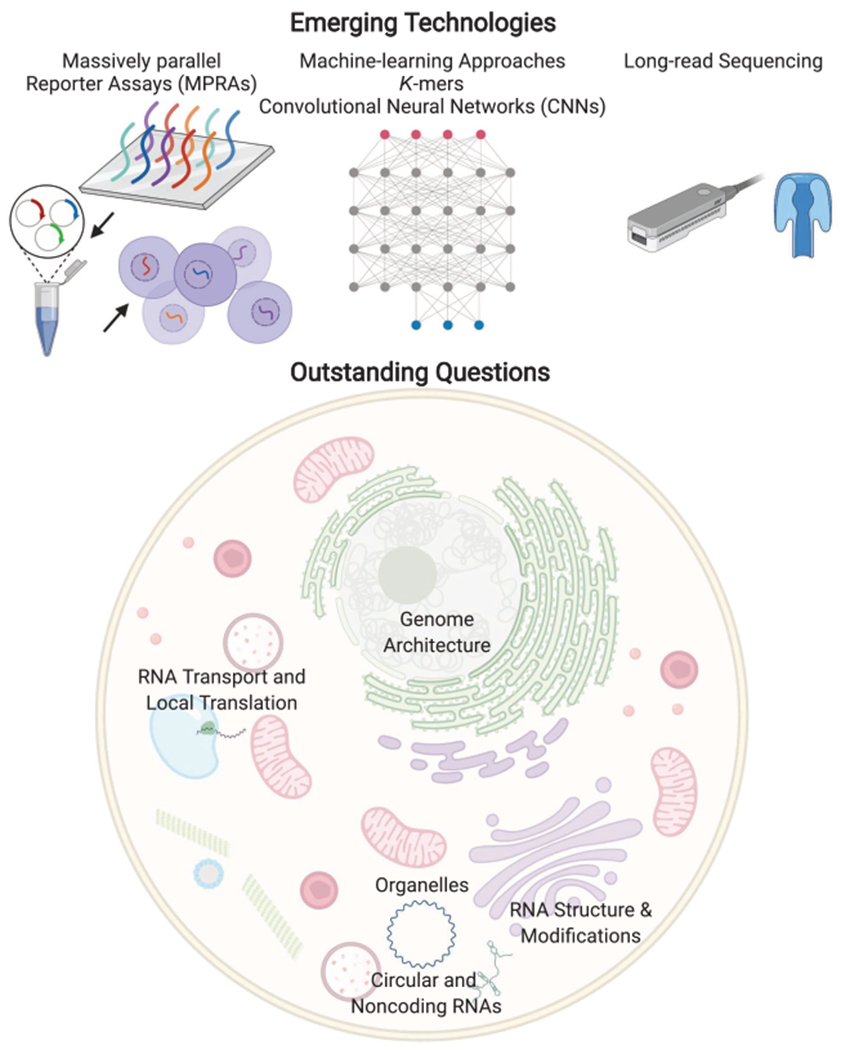
The latest approaches and outstanding questions in investigating RNA localization.

Subcellular RNA-seq data, including from the nucleus, cytosol, and the ER, provide rich data sets to identify the sequencing elements involved in RNA localization. Applying machine-learning approaches, including deep-learning algorithms (LeCun et al. 2015), to these data sets is likely to provide new insights into the sequence-determinants of localization (Fig. 6). For example, bioinformatics approaches have identified transposable elements as being important for the nuclear retention of many lncRNAs (Carlevaro-Fita et al. 2019). Furthermore, a statistical analysis has shown that the transposable element Alu has a strong preference for being in the 3′ UTR of transcripts that are overrepresented in the nucleus, Golgi, and mitochondria (Chen et al. 2018). Similarly, a deep-neural-network approach to predict lncRNA localization as nuclear or cytosolic directly from transcript sequences had modest success, approaching an accuracy of 72% (Gudenas and Wang 2018). In that study, the feature set for learning included sequences as *k*-mers, known RNA-binding protein motif sites, as well as genomic characteristics of the RNAs such as whether they were intergenic, antisense, or sense lncRNAs. Likewise, another group used *k*-mers along with other features to obtain an accuracy of 59% for localization of lncRNAs (Cao et al. 2018), although another group in a different context claimed 87% accuracy using 8-mer nucleotide segments along with other features (Su et al. 2018). Another model called RNATracker has used a convolutional neural network to classify RNA localization; so far, success has been modest (Yan et al. 2019). A recent computational approach called RNA-GPS (Wu et al. 2020), which uses an APEX-seq data set (Fazal et al. 2019) comprising the subcellular transcriptome of eight locations, uses *k*-mers as features to obtain an overall accuracy of 70%. RNA-GPS implicates transcript splicing as an important process influencing localization for organelles within both the nucleus and cytosol. In summary, although such approaches are in their infancy, they should provide candidates sequences that can be directly tested for their localization potential, and the corresponding interacting RBP identified (Wu et al. 2020).

### Massively Parallel Reporter Assays

An experimental strategy to identify and test for zip code sequences within cells is the use of massively parallel reporter assay (MPRAs), in which tens of thousands of sequences, typically 75–200 nt in length, can be interrogated. Using MPRAs, along with machine-learning models, particularly convolutional neural networks (CNNs) (Movva et al. 2019), is likely to facilitate the rapid discovery of zip code sequences. Two groups recently used high-throughput screens to identify *cis*-acting RNA localization elements that promote nuclear retention. Rinn and coworkers tested and designed more than 10,000 oligos derived from 38 human lncRNAs with known both nuclear and cytosolic localization. Similarly, the Ullitsky group used approximately 5500 oligos gathered from 37 lncRNAs as well as some mRNAs. Both these studies were performed by introducing these oligos into an RNA and assessing its change in nuclear retention by nuclear-cytosolic fractionation following by sequencing. Through the MPRA experiments, the Ulltisky group found a cytosine-rich element, RCCTCCC (R = A/G), derived from an antisense Alu element, which they named SIRLOIN (SINE-derived nuclear localization element), that promotes nuclear retention (Lubelsky and Ulitsky 2018). By screening the binding sites of more than 100 RBPs using publicly available RBP-binding data sets (Van Nostrand et al. 2016), they identified the heterogeneous nuclear ribonucleoprotein K (HNRNPK) as binding to and nuclear-retaining SIRLOIN-containing RNAs. The Rinn group found a similar motif contributing to nuclear retention. The role of SINE elements in nuclear retention of *MALAT1* lncRNA through HNRNPK recruitment was recently confirmed by another study (Nguyen et al. 2020).

MPRA-based screens are likely going to be a powerful way to screen for zip code components, including sequence motifs and structural elements. Concomitantly, MPRA experimental and computational strategies continue to improve, and it is now possible to test more than 100 million sequences (de Boer et al. 2019).

### Long-Read Sequencing

Next-generation-sequencing (NGS) approaches, including using the Illumina platform, continue to transform in biology. However, a significant limitation continues to be the relatively short sequencing reads (typically <300 base-pair [bp]) generated. Fortunately, the latest third-generation approaches such as Oxford Nanopore Technologies (ONT) and Pacific Biosciences (PacBio) provide much longer-read sequencing reads (>1000 bp) and can sequence RNA directly without having to reverse transcribe it to make complementary DNA (cDNA) (Garalde et al. 2018). By being able to generate full-length transcript sequences, in addition to yielding RNA modification (Soneson et al. 2019; Workman et al. 2019), these techniques can reveal the landscape of variation in splicing isoforms, poly(A)-tail-length (Legnini et al. 2019), and RNA modifications (Workman et al. 2019). Exciting future studies will undoubtedly implement these approaches to explore transcript-isoform localization differences and dissect the role of RNA modifications in localization. Previous studies indeed identify the abundant N6-methyladenosine (m^6^A) modification to be important for facilitating the nuclear export of mRNAs, with modified transcripts “fast-tracked” to the cytosol for translation (Lesbirel and Wilson 2019).

## SOME OUTSTANDING QUESTIONS IN THE FIELD

Although RNA localization studies have a rich history spanning more than three decades, many critical issues in the field remain unanswered. The central question continues to persist: Why do cells localize their RNA contents? In some cell types, such as neuronal cells in which the distances involved for transporting biomolecules are vast, it is easy to rationalize that actively transporting mRNAs to their destination to be locally translated to make proteins would be convenient and efficient. However, it remains unclear why RNA subcellular localization is ubiquitously observed in almost all cell types, including ones in which the process of diffusion should be fast (seconds or less). To address the question of why cells localize their RNA contents, we must first explain the following questions.

### Relative Contribution of Translation- versus RNA-Dependent mRNA Localization

A vital issue in the field is ascertaining to what extent the observed subcellular RNA localization is translation-dependent, and whether RNAs can be transported, particularly actively by molecular motors, with the ribosome engaged in the translation of the mRNA. It was generally accepted that the transport of mRNAs occurs through mRNPs that are translationally repressed until they get to their destination (Fig. 6). Furthermore, translating mRNAs interact with RNP granules dynamically, whereas nontranslating mRNAs can form stable associations (Moon et al. 2019). However, recent studies have begun to question this assumption, including imaging experiments that have revealed that active transport of mRNAs can occur after the mRNA has started translation and entered the polysome state (Wang et al. 2016; Moon et al. 2019). Similarly, APEX-seq has revealed that many nuclear RNAs destined for the mitochondria begin the process of translation elsewhere, such as in the cytosol, and then the translating-ribosome complex comprising of the nascent peptide being synthesized, RNA, and ribosome is directed to the mitochondria. APEX-seq experiments (Fazal et al. 2019) also implicated the cytoskeleton and its associated motors as being necessary for this transport, suggesting that engagement of mRNA with transport motors and translating ribosomes can co-occur. These studies also indicate that some observed mRNA localization is a consequence of translation, as has been suggested for mitochondria in yeast (Eliyahu et al. 2010). In contrast, other RNAs were found to localize to the mitochondria independent of translation and to be preferentially coding for mitoribosome and oxidative phosphorylation proteins.

Understanding how RNAs find their destination continues to be a fascinating problem that will require imaging, sequencing, and biophysical insights. Cells rely on different approaches to transport mRNAs, and future studies will likely also focus on understanding how organelles are optimized and regulated to control the localization of transcripts to them (Tsuboi et al. 2019).

### Role of RNA Modifications and Structure

RNAs are extensively modified within cells, and there exist more than a hundred types of chemical modifications (Roundtree et al. 2017a), some of which are likely to be important in specifying RNA localization. For example, the abundant epitranscriptomic modification m^6^A has been shown to influence the nuclear export of RNAs, with the m^6^A-binding protein YTHDC1 mediating this process (Roundtree et al. 2017b). RBPs such as FMRP have been identified as m^6^A readers that promote export (Edens et al. 2019), and RNA modifications are known to be involved in forming and localizing to phase-separated, membrane-less granules under stress conditions. Furthermore, m^6^A-modified mRNAs are enriched in stress granules (SGs), and the m^6^A-binding YTHDF protein is critical for SG formation (Fu and Zhuang 2019; Ries et al. 2019). Likewise, changes in the poly(A)-tail length at the end of 3′ UTRs have been implicated with RNA-localization changes (Fazal et al. 2019). Thus, although evidence for widespread involvement of modifications in RNA localization remains limited, these multiple observations in different systems warrant future investigation.

In addition to RNA modification, the secondary and tertiary structures of RNAs undoubtedly guide RNA localization patterns. RNA structure within cells varies across different cellular locations (Sun et al. 2019), and many RBPs such as Staufen interact with structural elements in RNAs (Bevilacqua et al. 2016). Furthermore, structured RNAs (Langdon et al. 2018; Maharana et al. 2018) in different subcellular locations show different propensities for forming liquid-liquid phase-separated condensates and organelles, including nuclear speckles, paraspeckles, Cajal bodies, nuclear stress bodies, and even heterochromatin (Sanulli et al. 2019). Thus structure-mapping studies should complement localization studies in identifying *cis* elements directing RNA localization.

### How RNAs Influence the Genome Architecture

Genomic DNA is highly organized in three-dimensional space, and RNA has long been known to be an essential regulator of chromatin (Nickerson et al. 1989). RNA binding seems to promote CTCF-dependent chromatin looping and thus is vital for the organization of the genome into megabase structures called topologically associated domains (TADs) (Saldana-Meyer et al. 2019). Furthermore, RNAse treatment to degrade RNAs, as well as transcriptional inhibition, affects both the structure and formation of DNATADS (Barutcu et al. 2019). Likewise, disruption of the RNA-binding domain of CTCF, including through mutations (Hansen et al. 2019), has a global effect on chromatin binding, gene expression, and the formation of chromatin loops. However, the exact identity of RNAs in each genomic neighborhood within the nucleus that modulates the underlying processes of transcription, splicing, and genome organization remains unclear. A recently developed technology to perform RNA-directed chromosome conformation may aid in solving this mystery (Mumbach et al. 2019).

Recent studies suggest RNAs can act as structural scaffolds for organizing chromatin domains, including the lncRNA *Firre* that maintains the H3K27m3 chromatin state of the inactive X chromosome in female cells and makes contact with several autosomes (Thakur et al. 2019). Other RNAs such as *MALAT1* and *NEAT1* have also been shown to have scaffolding roles within the nucleus, particularly within the nuclear speckles and paraspeckles respectively. Another RNA*Xist*, required for transcriptional silencing of the X chromosome, is brought to the nuclear lamina as part of its function (Chen et al. 2016).

APEX-seq in the nucleus revealed a correlation between the location of mature, polyadenylated transcripts, and the underlying genome architecture (Fazal et al. 2019). For example, the lamina transcriptome was found to be enriched for genes found in lamina-associated domains (LADs), and the nucleolus transcriptome is enriched for genes found in nucleolus-associated domains (NADs). LADs, DNA regions near the lamina, comprise 30%–40% of the genome and contain thousands of genes that are generally lowly expressed. In summary, there seems to be an intimate connection between subnuclear RNA localization and the underlying genome organization and regulation that warrants further investigation.

### How RNAs Localize to Organelles

How cells orchestrate the localization of hundreds of RNAs to a subcellular location continues to remain a mystery. Locales such as the ERM and OMM are known to have more than a thousand transcripts localizing there. Recently, APEX-seq (Fazal et al. 2019) revealed the landscape of RNA localization and local translation to the outside of the mitochondria, identifying both translation-dependent and translation-independent mechanisms of RNA localization. For reasons not clear, the translation-independent transcripts had shorter 3′ UTRs and shorter poly(A)-tail lengths. Similarly, the RBP CLUH is known to bind a subset of mRNAs for nuclear-encoded mitochondrial proteins in mammals (Gao et al. 2014). Nonetheless, the localization mechanism of transcripts to the mammalian mitochondria remains opaque and will undoubtedly be an active area of future investigation.

In yeast, where RNA localization to the mitochondria is better understood, it has been speculated that the mitochondrial proteins translated near the mitochondria are of prokaryotic origin, whereas accessory proteins are often translated in free cytoplasmic polysomes (Garcia et al. 2007; Marc et al. 2002). Furthermore, although the localization of proteins to the mitochondria is aided by specific amino acids in the translated nascent peptide, called mitochondria-targeting sequences (MTSs), sequences in the 3′ UTR of the corresponding RNA have also been shown to be essential for local translation. For example, in yeast, either the MTS or the 3′ UTR was sufficient to independently target ATM1 mRNA to the vicinity of the mitochondria (Corral-Debrinski et al. 2000). Also, some RBPs such as the Puf family of proteins in yeast control the localization of hundreds of transcripts, particularly Puf3 that associates with transcripts encoding proteins localizing to the mitochondria (Hogan et al. 2008).

In addition to the ER and mitochondria, many locations in cells concentrate RNAs, including MLOs present in both the nucleus and cytosol. Interestingly, the MLOs’ nucleolus and stress bodies are known to phase separate and are tuned and regulated by the concentration of proteins and RNAs within them. Furthermore, long RNAs with stable secondary structures that bind RNA binding proteins are particularly good at promoting phase separation, including in nuclear locations such as paraspeckles. Understanding how RNAs are specifically targeted to MLOs and membrane-bound organelles continues to be a fascinating, unanswered question.

### How Nonpolyadenylated RNAs, including Circular RNAs, Localize within Cells

Cells contain many different RNA species, and extending our current understanding of mRNA localization to other RNA species, including tRNAs and circular RNAs (circRNAs), will be important in understanding the regulation of these molecules. circRNAs have received a lot of interest in recent years, and it is known that they can code for proteins (Jeck and Sharpless 2014) and show asymmetric subcellular localization (Saini et al. 2019). Initial studies, for example, suggest circRNAs localize differently relative to other RNAs in neuronal projections (Saini et al. 2019). In addition, immunogenic circRNAs that are sensed as foreign are localized to distinct locations in the cytoplasm compared to endogenous circRNAs (Chen et al. 2019b). Thus, the mechanisms of circRNA localization, often without the benefit of 5′ or 3′ UTRs present on linear mRNAs, are likely to shed new light on RNA localization and circRNA functions.

## CONCLUSION

Subcellular RNA localization is an essential but underappreciated aspect of gene regulation. This review focuses on the eukaryotic cell, but even prokaryotic cells are known to have highly localized RNAs (Nevo-Dinur et al. 2011). In prokaryotes, RNAs are directed to specific locations such as the inner membrane, although whether this localization is exclusively translation-dependent or not remains an open question (Moffitt et al. 2016). With the advent of high-throughput imaging and sequencing approaches, it is now possible to comprehensive interrogate the transcriptomes of subcellular locations in different cell types and model systems. Exciting future studies will undoubtedly map out the regulatory code guiding localization, and explain why organisms ubiquitously use such mechanisms.
